# Report on the development of putative functional SSR and SNP markers in passion fruits

**DOI:** 10.1186/s13104-017-2771-x

**Published:** 2017-09-06

**Authors:** Zirlane Portugal da Costa, Carla de Freitas Munhoz, Maria Lucia Carneiro Vieira

**Affiliations:** Departamento de Genética, Escola Superior de Agricultura “Luiz de Queiroz”, Universidade de São Paulo, 13418-900 Piracicaba, Brazil

**Keywords:** *Passiflora*, Passionflowers, Molecular polymorphism, Microsatellites, Single nucleotide polymorphisms, Pathogen induced transcripts

## Abstract

**Background:**

Passionflowers *Passiflora edulis* and *Passiflora alata* are diploid, outcrossing and understudied fruit bearing species. In Brazil, passion fruit cultivation began relatively recently and has earned the country an outstanding position as the world’s top producer of passion fruit. The fruit’s main economic value lies in the production of juice, an essential exotic ingredient in juice blends. Currently, crop improvement strategies, including those for underexploited tropical species, tend to incorporate molecular genetic approaches. In this study, we examined a set of *P. edulis* transcripts expressed in response to infection by *Xanthomonas axonopodis*, (the passion fruit’s main bacterial pathogen that attacks the vines), aiming at the development of putative functional markers, i.e. SSRs (simple sequence repeats) and SNPs (single nucleotide polymorphisms).

**Results:**

A total of 210 microsatellites were found in 998 sequences, and trinucleotide repeats were found to be the most frequent (31.4%). Of the sequences selected for designing primers, 80.9% could be used to develop SSR markers, and 60.6% SNP markers for *P. alata*. SNPs were all biallelic and found within 15 gene fragments of *P. alata*. Overall, gene fragments generated 10,003 bp. SNP frequency was estimated as one SNP every 294 bp. Polymorphism rates revealed by SSR and SNP loci were 29.4 and 53.6%, respectively.

**Conclusions:**

*Passiflora edulis* transcripts were useful for the development of putative functional markers for *P. alata*, suggesting a certain level of sequence conservation between these cultivated species. The markers developed herein could be used for genetic mapping purposes and also in diversity studies.

**Electronic supplementary material:**

The online version of this article (doi:10.1186/s13104-017-2771-x) contains supplementary material, which is available to authorized users.

## Background

Several tropical fruit species are underexploited but have promising economic potential for both industrial processing and *in natura* consumption. In Brazil, passion fruit is widely cultivated and the most recent survey on agricultural production showed that approximately 58,089 hectares were planted and 838,244 tons of fruit harvested [1]. Commercial plantations are almost exclusively based on a single species, the yellow passion fruit (*Passiflora edulis*), which is cultivated in over 90% of orchards. It is grown for its edible and aromatic fruits used in juice concentrate blends that are consumed worldwide. A second species, the sweet passion fruit (*P. alata*) is native to the Brazilian plateau and the eastern Amazonian region, but cultivated fairly widely only in the South and Southeast of Brazil, as reported in Cerqueira-Silva et al. [2]. It is fairly popular for its typical aroma and flavor characteristics, and therefore can fetch up to three times the price of the yellow passion fruit at local fresh fruit markets. Moreover, the vines of *P. alata* contain bioactive compounds [3] which have been widely used in a traditional medicine, and include a natural sedative, passiflorin.

*Passiflora edulis* and *P. alata* belong to the family Passifloraceae, order Malpighiales. Both are self-incompatible [4, 5], diploid species (*2n* = *18*) [6, 7] with perfect, insect-pollinated flowers. Despite the importance of *P. edulis* as a fruit crop, long-term breeding programs are still in the early stages. On the other hand, the sweet passion fruit is gaining importance for its attractive flowers and exotic flavored fruits, but no commercial varieties are available to farmers. Furthermore, the incidence of diseases can inflict losses in plantations of both species. For instance, bacterial spot caused by *Xanthomonas axonopodis* pv. *passiflorae* (*Xap*) harms both leaves and fruits and has no effective chemical control [8]. It is therefore very difficult to manage.

Currently, plant research tends to incorporate molecular marker-based approaches into conventional breeding programs, including programs for understudied tropical species. In particular, molecular markers derived from DNA sequences characterized as microsatellites or simple sequence repeats (SSRs), and single nucleotide polymorphism (SNPs) located in plant genes have been developed [9, 10] and used to reach the objectives [11, 12]. With this in mind, we used the transcripts from a subtractive library enriched for *P. edulis* genes expressed in response to *Xap* infection [13] to develop putative functional SSR and SNP markers for *P. alata*.

## Methods

### Origin of the DNA sequences

The aim of the study was to develop a set of functional markers for *P. alata* from *P. edulis* transcript sequences induced and repressed in response to *Xap,* based on two SSH (suppression subtractive hybridization) libraries previously constructed in our laboratory. The forward (F) library was enriched for cDNA fragments of genes whose expression level increased following bacterial inoculation. Conversely, in order to isolate cDNA fragments of genes whose expression level decreased following inoculation, a reverse subtraction (R) was carried out with cDNA from mock-inoculated plants used as ‘testers’ and cDNA from inoculated plants used as ‘drivers’ [14]. We were able to isolate 683 transcripts from the forward (F) library, 274 from the reverse (R) library, and 41 transcripts referred to contigs of sequences derived from both libraries [13], giving a total of 998 sequences that were used for developing putative functional markers as described below.

### Plant material and DNA isolation

Two accessions of *P. alata* and two of *P. edulis* were used to develop the SSR markers. These accessions were the respective parents of full-sib progenies used for mapping purposes in our laboratory [15, 16]. *P. alata* accession ‘2(12)’ belongs to a progeny of a wild ancestor that was collected in a locality between the Brazilian Amazon and Cerrado ecosystems [17]. *P. alata* accession ‘SV3’ is an indoor selection cultivated in the Southeast of Brazil. Both *P. edulis* accessions belong to the germplasm collection of The Agronomic Institute of Parana State, Brazil. ‘IAPAR-123’ is a selection from the Brazilian commercial population used for industrialized juice production and ‘IAPAR-06’ was introduced from Morocco.

All plant genomic DNA was extracted from young leaf tissue using the cetyltrimethylammonium bromide method adapted from Murray and Thompson [18]. For DNA quantification, samples were electrophoresed in 1.2% agarose gels stained with SYBR SAFE^®^ (Invitrogen, Carlsbad, CA, USA), with 1× TBE as the running buffer, and compared to known concentrations of λ phage DNA. The gels were visualized under UV light and photo documented by a UVP MultiDoc-It digital imaging system. The original samples were diluted to 10 ng µ1^−1^.

After testing the ability of *P. edulis* SSR primer pairs to generate reproducible amplicons in *P. alata*, the loci that revealed polymorphisms between the ‘2(12)’ and ‘SV3’ accessions were used for genotyping 30 individuals of the segregating progeny (F_1_). For developing SNP markers, the same parental accessions of *P. alata* and two F_1_ genotypes (‘F_1_-67’ and ‘F_1_-100’) were used for prospecting sequence polymorphisms as explained below.

### Microsatellite prospection and development of SSR markers

MISA software [19] was used to search for microsatellites in both the F and R libraries cited above. The following parameters were used to identify microsatellites: mono-, di-, tri-, tetra-, penta- and hexanucleotide repeats with a minimum of 10, 5, 3, 3, 3 and 3 subunits, respectively. SSRs were grouped according to motif, and each group included all the complementary and permuted sequences. SSR markers were developed from a set of genes selected from the F library [13].

*Passiflora edulis* selected transcripts containing SSRs were analyzed for primer designing. The PRIMER3 [20] and Gene Runner [21] computational programs were used. Next, amplification reactions were performed as follows: 30 ng genomic DNA, 1× PCR buffer, 0.25 mM dNTP, 0.3 µM of each primer, 2 mM MgCl_2_, and 1.2 U Taq DNA polymerase (Promega, Madison, WI, USA). Ultrapure water was added to make up the volume to 20 µl. Cycling consisted of an initial denaturing step of 5 min at 95 °C, 30 cycles of 40 s at 95 °C, 40 s at 55 °C, 60 s at 72 °C and a final extension of 8 min at 72 °C. PCR products were electrophoresed in 1.2% agarose gels stained with SYBR SAFE^®^ (Invitrogen), with 1× TBE as the running buffer, and compared to the 100 bp DNA Ladder (Invitrogen). PCR conditions were optimized where necessary and adjusted depending on the amplification patterns. Note that tests at different primer concentrations combined with specific annealing temperatures were carried out.

For polymorphism evaluation, DNA samples were first denatured for 5 min at 95 °C and rapidly ice cooled. For each sample, 2.5 µl was loaded on a polyacrylamide gel (0.4 mm) and the products separated by denaturing electrophoresis. The electrophoresis was run in a vertical system (Sequi-Gen^®^ GT Sequencing Cell, Bio-Rad, 38 × 50 cm gels). The gels contained 5% 19:1 acrylamide/bisacrylamide, 7 M urea and 1× TBE, and were run at 80 W for 2 h 30 min at 45° C. The glass plate with the gel firmly adhered to it was rinsed thoroughly in order to stain the DNA bands with silver nitrate (0.2%). Amplicons were visualized after adding sodium carbonate according to Creste et al. [22]. The gels were then analyzed under white light and digitized (Epson Expression 10,000 XL). The genetic configuration at each SSR locus was analyzed as proposed by Wu et al. [23].

### Development of SNP putative markers

A total of 122 transcript sequences from *P. edulis* were selected for designing the primers (Additional file 1: Table S2). Primer sequence designing, amplification reactions and cycling programs, as well as amplicon electrophoresis were performed as described for SSR marker development. Amplifications using *P. alata* DNA that resulted in specific bands were purified using Illustra GFX 96 PCR Purification Kit^®^ (GE Healthcare, Little Chalfont, BUX, UK). Concentrations of the purified products were estimated by spectrophotometry using a NanoDrop 2000^®^ (Thermo Scientific, Waltham, MA, USA), and purification was checked by electrophoresis in 1.2% agarose gels stained with SYBR SAFE^®^ (Invitrogen), with 1× TBE as the running buffer, and compared to phage λ DNA of known concentrations.

For sequencing reactions, the BigDye^®^ Terminator v3.1 Cycle Sequencing Kit (Applied Biosystems, Carlsbad, CA, USA) was used. Each reaction contained 50 ng of purified PCR product, 2 µl of 5× buffer, 0.6 µl of BigDye Terminator v3.1, 1.0 µl of each primer (forward or reverse), and ultrapure water to make up to a total volume of 10 µl. The following cycling program was used: 1 min at 96 °C, followed by 25 cycles of 10 s at 96 °C, 5 s at 50 °C and 4 min at 60 °C. Capillary electrophoresis was run on an ABI 3730 DNA Analyzer^®^ (Applied Biosytems).

We routinely sequenced the positive strand (forward) of each genotype DNA but occasionally the negative strand (reverse) was also sequenced. Sequence data were processed using the CodonCode Aligner^®^ 3.7.1 (CodonCode Corporation, Centerville, MA, USA), a base-calling software which assigns a quality phred-based score to each base [24]. Low quality bases (phred < 20) were trimmed. Sequences were then aligned with each other using the assemble function to identify polymorphisms. A single position in DNA sequences with a nucleotide variation was considered polymorphic. The parental genotypes and two F_1_ genotypes (F_1_-67 and F_1_-100) of *P. alata* were compared.

## Results and discussion

**Fig. 1:**
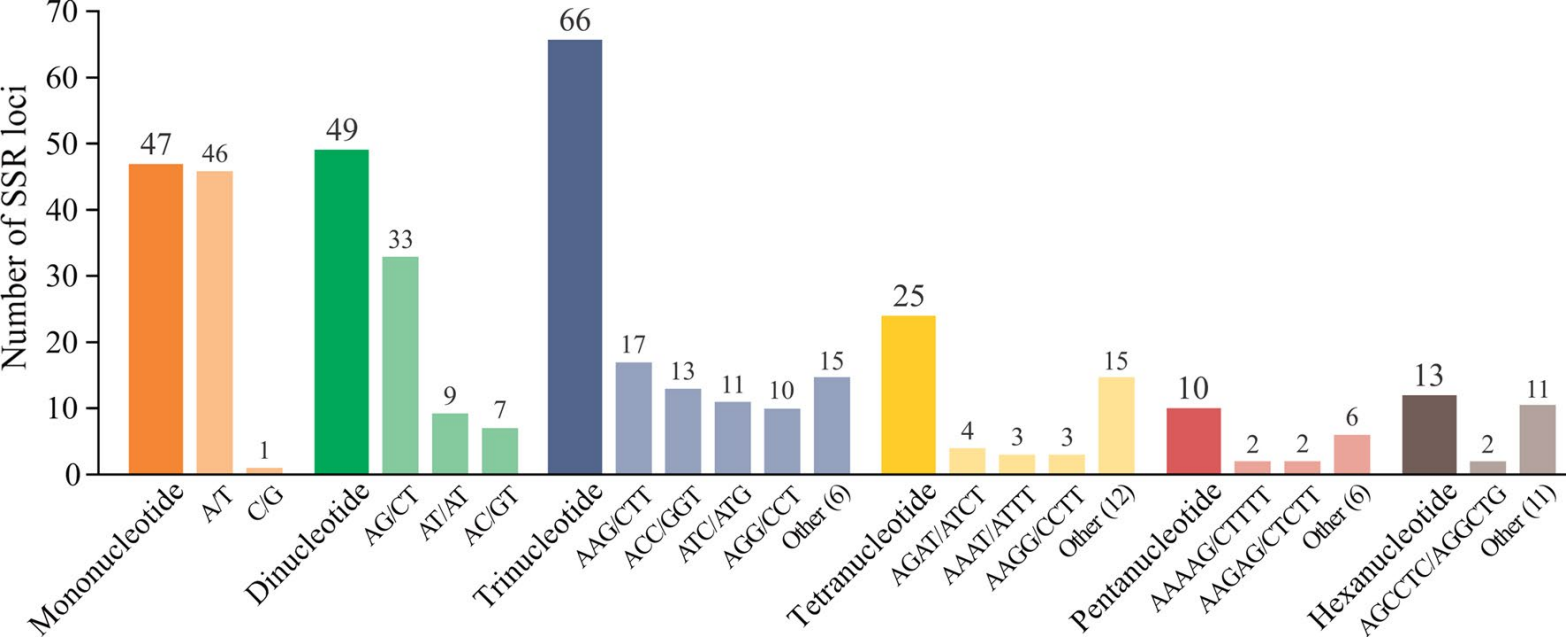
Percentage of mono-, di-, tri-, tetra-, penta- and hexanucleotides in the microsatellites found in transcribed sequences of *Passiflora edulis* (Passifloraceae); the percentage of the most common motif is shown for each case

We identified 210 SSRs in 998 *P. edulis* transcript sequences (21.04%), including 22.4% mono-, 23.3% di-, and 31.4% trinucleotide repeats. The remaining 11.9, 4.8 and 6.2% were tetra-, penta- and hexanucleotides. The nucleotide composition of the repeat motifs showed that A/T (98%), AG/CT (67.3%) and AAG/CTT (25.7%) were the most abundant among the mono-, di- and trinucleotides (Fig. 1). In terms of microsatellites (SSRs), 95% were perfect, 2% interrupted, 3% imperfect and 1.5% compound.

These results are in line with other studies on Malpighiales species, such as *Salix* spp. and *Ricinus communis*, in which trinucleotide repeats were the most abundant class in expressed sequences [25, 26]. Some other studies have shown different results, such as those on *Manihot esculenta* [27]. The variation found in SSR frequency is plausible based on the variation in sample size, search criteria, database size and tools used for the SSR development, as well as intrinsic differences between the species evaluated [28]. Trinucleotides and perfect microsatellites were expected to be predominant, since the SSRs examined were within coding sequences. In terms of nucleotide composition, our results are in line with those reported for Malpighiales, such as *Populus euphratica* [29] and *Jatropha curcas* [30].

**Fig. 2:**
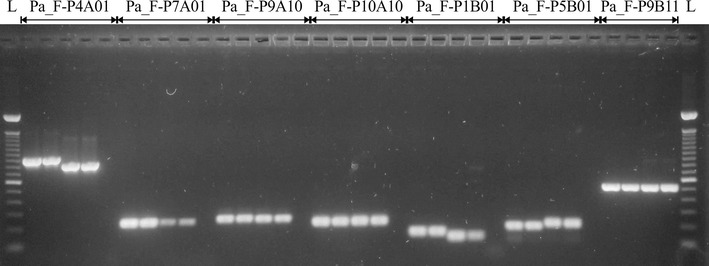
Amplification patterns of 7 SSR loci in *Passiflora edulis* (*first two lanes*, accessions ‘IAPAR-06’, ‘IAPAR-123’) and *P. alata* (*third and fourth lanes*, accessions ‘2(12)’, ‘SV3’) following agarose gel (1.2%) electrophoresis at 3 V cm^−1^ for 2 h. *Lane L*, 100 bp ladder

In all, 41 out of 42 primer pairs designed from selected sequences of *P. edulis* resulted in amplification. Compared to *P. alata*, after optimizing PCR conditions, we were able to obtain good amplification patterns for most of the primer pairs (Fig. 2), revealing high transferability rates between *P. edulis* and *P. alata* of approximately 80.9% (34/42).

Silva et al. [31] investigated the transferability of 24 primer pairs developed for *P. edulis* and 7 for *P. alata* to wild species of *Passiflora* (*P. cacao*, *P. cincinnata*, *P. glandulosa*, *P. gibertii*, and *P. mucronata*). The interspecies cross-amplification rate varied, and the lowest was 14.4% between *P. alata* and *P. cincinnata*. Transferability from *P. alata* and *P. edulis* to *P. cacao* was the highest, at 28.5 and 62.5% respectively. In general, the transferability of SSR loci was successful.

Our study shows that genic SSR locus transferability between *P. edulis* and *P. alata* is high. In fact, *P. edulis* and *P. alata* belong to the same subgenus (*Passiflora*), though to different botanical supersections (*Passiflora* and *Laurifoliae* respectively), and series (*Passiflora* and *Quadrangulares*, respectively). Interspecific hybrids of the two species are only 5% fertile [32].

As mentioned above, *P. edulis* and *P. alata* are self-incompatible species [4, 5] with an obligatory allogamous mode of reproduction, posing difficulties in the production of inbred lines, which are routinely used to generate hybrids that do not segregate. In the case of outcrossing species, which is enforced by self-incompatibility in *P. edulis* and *P. alata*, the F_1_ populations (or full-sib progenies) do segregate [15, 16, 23]. Therefore, after testing the ability of *P. edulis* SSR primer pairs to generate reproducible amplicons in *P. alata*, the loci that revealed polymorphisms between the parental accessions were used for genotyping individuals of the segregating progeny (F_1_) as reported below.

**Fig. 3:**
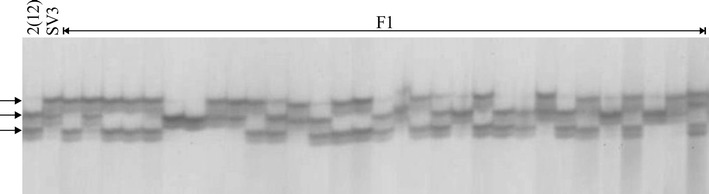
SSR locus (Pa_F-P5B01) segregating in an F_1_ population of *Passiflora alata* (N = 30). Parental accessions [‘2(12)’ and ‘SV3’] are shown in the *first and second lanes*. The locus configuration allowed the identification of three alleles (*arrowed*), and four genotypes. Polyacrylamide gel (1.2%) electrophoresis run at 80 W for 3 h

Herein, the PCR fragments obtained using 34 primer pairs were separated in denaturing polyacrylamide gels and 10 fragments revealed 15 polymorphic loci in *P. alata* (Table 1). Loci Pa_F-P5B01, Pa_F + R-Contig114, Pa_F-Contig13 and Pa_F-P8E05 showed four alleles. According to Wu et al. [23], these are the most informative loci for generating a high-resolution map, which would require four alleles segregating in a 1:1:1:1 ratio in F_1_ (when both parents are heterozygous for different alleles), and this configuration is possible only using SSR markers. For the remaining 6 loci, two alleles were identified (Table 1; Fig. 3).

**Table 1:** Genetic configuration of 10 SSR putative functional markers developed for *Passiflora alata*

SSR marker code	Primer sequences (5′–3′)	Annealing temperature (°C)	Locus configuration^a^	Observed segregation in the F_1_ population (N = 30)^a^	Number of alleles
Pa_F-P10A10	F:TGGAATGTGATTTGCATGG R:TAGGTATTGCTGGCGAAG	55	1 (*oo* × *ao*)	1:1	2
Pa_F-P5B01	F:GCGATGAGTGTGCTAGAGG R:TTCAGACAGCCGAGGAAG	55	1 (*ab* × *cd*)	1:1:1:1	4
Pa_F-Contig77	F:AAGCCAAAGTCGGTGTTG R:TAGCCCAGACAAGGAAGG	60	1 (*oo* × *ao*) 2 (*oo* × *ao*)	1:1 1:1	2 2
Pa_F + R-Contig114	F:CTTGCCTCTCTCCCTCTC R:GGGTTTTGGGTTTGACAG	58	1 (a*o* × *bo*)	1:1:1:1	4
Pa_F-Contig13	F:CAGAAGGAAAACCAGCAAG R:CCCCATCATCATCACTTTC	60	1 (a*o* × *bo*)	1:1:1:1	4
Pa_F-Contig66	F:TCCCCCATTTTCTCTTCG R:CGATTCCCGAACAAGTAAG	55	1 (a*o* × *bo*)	1:1	2
Pa_F-P7E10	F:TTAATGCCACAGCCCAAC R:TGACCCAAAATCAAACACC	55	1 (*oo* × *ao*)	1:1	2
Pa_F-P8E05	F:GGAAGCAAACACCAAAATC R:TCGTCGTCATCGAACCTC	55	1 (a*o* × *oo*)	1:1:1:1	4
Pa_F + R-Contig80	F:TGTTCAAGCCCATCTTCG R:GCTCTCGCTCTTGTCGTAG	55	1 (a*o* × *oo*) 2 (a*o* × *oo*) 3 (*oo* × *ao*) 4 (*oo* × *ao*)	1:1 1:1 1:1 1:1	2 2 2 2
Pa_F-P6H04	F:GAATAGGGTCAGCAGGAGG R:GAGTGTGTCATCGGAGTCC	55	1 (*oo* × *ao*) 2 (a*o* × *oo*)	1:1 1:1	2 2

^a^According to Wu et al. [22]

It is worth noting that all SSRs developed herein are putative functional markers that can be deployed in future genetic studies. They are within gene sequences that encode for distinct proteins (Table 1) (Additional file 1: Tables S1, S3). This is the first report on the development of genic SSR markers for *P. alata*.

In terms of SNP prospection, 74 primer pairs complementary to sequences of *P. edulis* resulted in unique amplicons in *P. alata* after performing optimized PCR assays. As expected, a moderate transferability rate between the species was observed (~60%, 74/122).

We selected 37 out of the 74 transcripts to obtain the Sanger sequences of genotypes ‘2(12)’, ‘SV3’, ‘F_1_-67’ and ‘F_1_-100’. Comparing the nucleotides that were aligned with each other, it was possible to detect at least one SNP in 15 gene sequences. SNPs were detected on the positive strand of 14 gene sequences in all genotypes, and only one SNP was detected on the negative strand of the Pa_F-P5A12 gene sequence (Table 2). The absence of SNPs was observed in 13 gene sequences, but the remaining 9 gene sequences were of relatively low quality, ruling out any analysis.

**Table 2:** SNPs found in *Passiflora alata* putative genes, their position in the contig, and the SNP class classified as intronic (I) and exonic (E)

SNP marker code	Position in the contig	Putative gene function	SNP class	Parental genotypes		F_1_ genotypes	
				‘2(12)’	‘SV3’	‘F_1_-67’	‘F_1_-100’
Pa_F-P9A06	532	Lipoxygenase [*Passiflora edulis*]	I	G/C	G/G	G/C	C/C
Pa_F-P5A12_F	127	ABC transporter G family member 36-like [*Brassica napus*]	E	A/T	T/T	A/T	T/T
	235		I	T/C	T/C	T/C	T/C
Pa_F-P5A12_R	160	ABC transporter G family member 36-like [*Brassica napus*]	I	G/T	G/T	G/T	G/T
	169		I	G/A	G/G	G/A	G/G
Pa_F-P9B08	333	KINASE 2B family protein [*Populus trichocarpa*]	I	A/A	A/C	A/A	A/A
	342		I	T/T	A/T	T/T	T/T
	344		I	C/C	A/C	C/C	C/C
	347		I	T/T	G/T	T/T	T/T
Pa_F-P10C11	44	ATP binding protein, putative [*Ricinus communis*]	E	?/?	C/T	C/T	C/T
Pa_F-Contig10	158	Uncharacterized protein LOC100855146 [*Vitis vinifera*]	E	G/C	G/G	G/C	G/C
Pa_F-Contig71	309	Hypothetical protein POPTR_0010s13740 g [*Populus trichocarpa*]	E	G/T	T/T	T/T	G/T
	323		E	C/C	G/C	G/C	G/C
Pa_R-Contig127	108	Hypothetical protein MANES_02G192700 [*Manihot esculenta*]	E	G/C	C/C	G/C	?/?
	336		I	G/A	G/G	G/A	?/?
	364		I	A/C	A/A	A/C	?/?
Pa_ F-Contig68	104	Unnamed protein product [*Vitis vinifera*]	E	T/C	T/C	T/C	C/C
	303		E	C/C	T/C	C/C	C/C
	343		E	T/C	C/C	?/?	T/T
	347		E	T/C	C/C	?/?	C/C
	349		E	T/C	C/C	?/?	T/T
Pa_F-P10D05	181	Hypothetical protein SOVF_097380 [*Spinacia oleracea*]	E	A/G	A/A	G/G	A/A
	350		E	A/T	T/T	A/A	T/T
	371		E	A/G	A/A	G/G	A/A
	381		E	A/G	G/G	A/A	G/G
Pa_F-P7E01	212	Hypothetical protein LR48_Vigan02g193200 [*Vigna angularis*]	E	G/C	C/C	G/C	G/C
Pa_F-P8F01	163	Syntaxin-32 [*Jatropha curcas*]	I	T/C	T/C	T/C	T/T
	246		I	T/G	T/G	T/G	T/T
	247		I	T/G	T/G	T/G	T/T
Pa_F-P4G06	287	Gamma-glutamylcysteine synthetase precursor [*Glycine max*]	I	G/C	C/C	C/C	C/C
Pa_F-P5G12	264	Hypothetical protein POPTR_0001s31350 g [*Populus trichocarpa*]	E	T/T	A/T	A/T	T/T
Pa_F-P9H12	286	Uncharacterized protein LOC105122267 isoform X2 [*Populus euphratica*]	I	G/A	G/A	G/A	G/A
Pa_R-P3A02	186	Hypothetical protein MANES_03G177700 [*Manihot esculenta*]	I	T/C	C/C	?/?	T/C
	225		I	T/C	C/C	?/?	T/C

SNP alleles were detected by evaluating the parental genotypes and two F_1_ genotypes

Sequence lengths ranged from 332 to 872 bp, with an average of 625 bp. There were differences between the observed and expected lengths for all 15 gene sequences. Thirteen were longer than predicted and three shorter. We designed primer pairs based on *P. edulis* transcripts and used them to amplify *P. alata* genomic DNA.

**Fig. 4:**
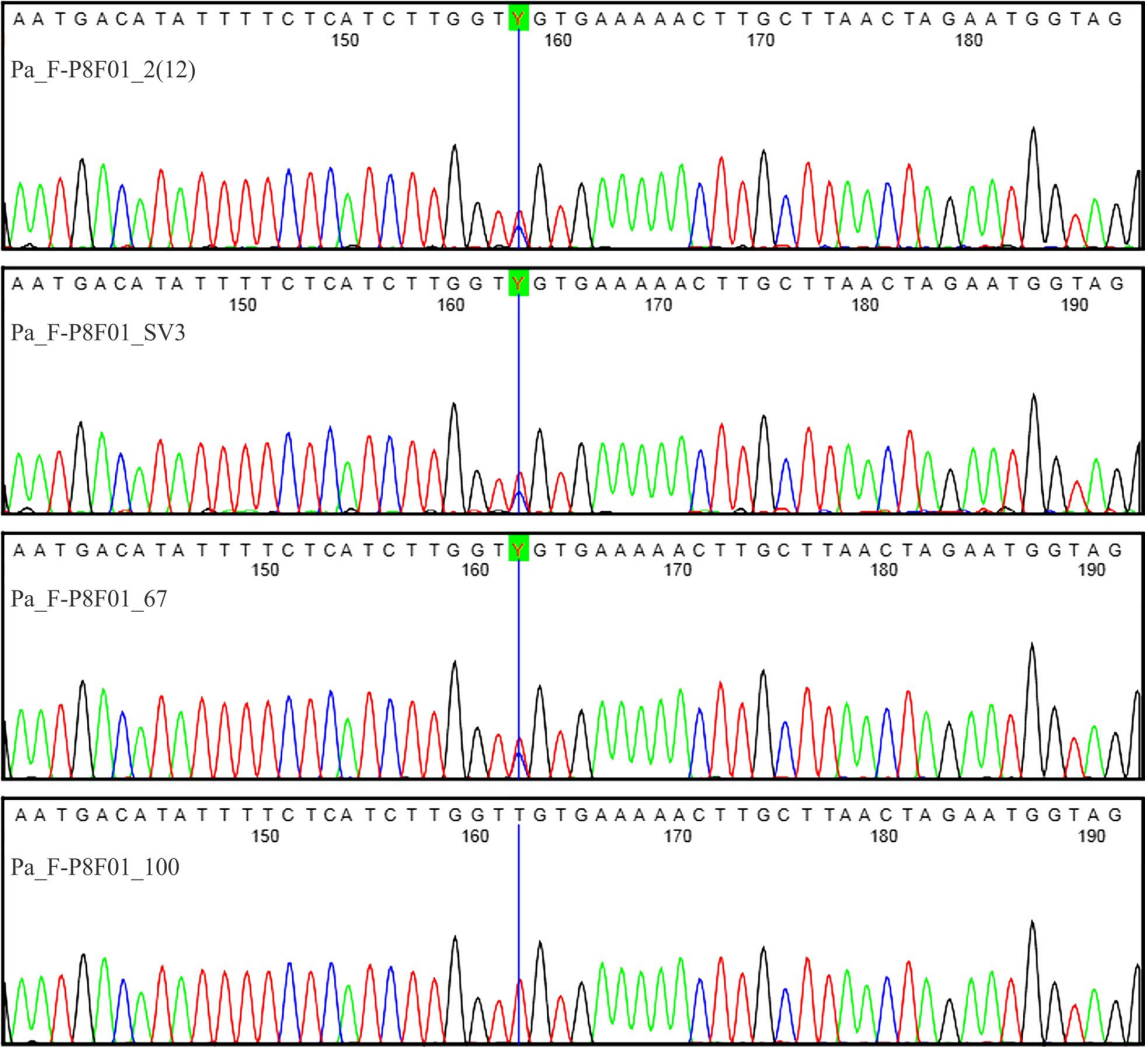
Alignment results for the Pa_F-P8F01 gene sequence showing an SNP at position 163

A total of 34 SNPs were identified in the 15 putative gene sequences of *P. alata*. We then used BLASTN to compare our results with the cDNA sequences of *P. edulis* and identify the position of every SNP in the genes, and find out whether they were located within introns or exons (Table 2). The alignment results of the Pa_F-P8F01 gene sequence are shown in Fig. 4.

SNPs were evenly distributed over coding and noncoding regions (i.e. 50%). Of the 17 SNPs found in exons, 13 (76.4%, 13/17) led to a change in the amino acid encoded, resulting in a 3.25:1 ratio (13/4) of non-silent to silent mutations. Five of the non-silent mutations were non-conservative, i.e. the nucleotide change resulted in the substitution of one amino acid for another with different properties that could impair protein function.

All nucleotide substitutions were biallelic. Transversions were slightly more common (53%, 18/34) than transitions (47%, 16/34). Transition mutations are expected to occur at higher frequencies than transversions. Furthermore, transitions are less likely to result in amino acid substitutions in protein sequences [33]. However, numbers can vary depending on sample size and selective pressure on the genes under analysis. For example, in *Manihot esculenta,* 52.6% (26,030/49,429) of SNPs were found to occur in coding sequences, 51.4% (40,561/78,854) were transition mutations and 48.6% (39,293/78,854) transversions [34], while in *Hevea brasiliensis*, 60% of SNPs (242,732/404,114) were transitions and 40% (161,382/404,114) transversions [35].

In *P. alata*, C/T and A/G transitions were predominant, with respective figures of 29.4% (10/34) and 17.6% (6/34). G/C transversions were predominant, accounting for 17.6% (6/34) of total transversions. Similarly, other studies have reported C/T or A/G as the most frequent transition mutations [35, 36].

It was possible to assign a putative function to six gene sequences. The remaining sequences (9) were found to be similar to hypothetical proteins (Additional file 1: Table S3). It is worth noting that the Pa_F-P9A06 locus sequence matches that of a lipoxygenase family protein (GenBank: ACS28586.1). Our group has recently reported that lipoxygenase 2 is highly implicated in *P. edulis* defense against *X. axonopodis* infection [13]. Similarly, the expression of the GhLOX2 gene was associated with the hypersensitivity response in cotton following inoculation with *X. campestris* pv. *malvacearum* [37]. It is therefore of interest to allocate the Pa_F-P9A06 locus to the linkage map of *P. alata* [16] as a beneficial complement to QTL (quantitative trait loci) mapping studies already performed in the same population of *P. alata* [38].

Overall, the 16 gene fragments generated 10,003 bp, so SNP frequency was estimated as approximately one SNP for every 294 bp. This frequency is lower than that found in a previous study (one SNP for every 177 bp) analyzing 7 genes in ‘2(12)’ and ‘SV3’ *P. alata* accessions [16]. These differences can be ascribed to the distinct set of genes analyzed. However, the SNP frequencies found herein are low in comparison to other outcrossing species [39–41]. Interestingly, the frequency of SNPs in *P. alata* putative genes is closer to that found in coding regions of self-pollinated species like *Arabidopsis thaliana* (1 SNP/336 bp) and rice (1 SNP/333 bp) [42, 43].

Finally, the polymorphism rates revealed by SSR and SNP loci were 29.4% (10/34) and 53.6% (15/28), respectively. The accessions did not contribute equally to polymorphisms. The contribution of the wild ‘2(12)’ accession was greater than that of the selected ‘SV3’ accession. In general, the polymorphism rates revealed by SSR markers were similar to those found in our previous studies using the same accessions of *P. alata*, with rates of 18% (308 AFLPs, amplified fragment length polymorphisms, and 20 SSR markers [44]), 20% (combination of 60 SSRs, 104 SNPs, 567 AFLPs, 12 TRAPs (target region amplification polymorphisms) and 23 RGAs (resistance gene analogs) [16]) and 35% (72 SSR markers [45]). Studying two distinct accessions of *P. edulis*, polymorphism rates were 26.4, 13.0 and 24.7% based on 113 RAPD (random amplified polymorphic DNA) [46], 174 AFLP [47] and 21 SSR loci [15], respectively. All these rates are considered low in view of the high levels of molecular polymorphism found in outcrossing species, like chicory (69,3%, based on 730 SSR markers [48]), cocoa (59%, based on 227 SSR markers [49]) and apple (50.5%, based on 338 SSR markers [50]).

## Conclusions

We were able to use gene sequences of *Passiflora edulis* to develop a set of genetic markers for *P. alata*, which can be used for genetic mapping purposes and also in diversity studies. A high to moderate level of transferability occurred between species (80.9% for SSR and 60.6% for SNP markers), providing tools for detecting polymorphic loci. We have provided additional information on the abundance of SSRs and SNPs in gene sequences of *P*. *alata*, and also confirmed the occurrence of low levels of molecular polymorphism in this outcrossing species.

## Additional file

**Additional file 1. Table S1:** Passion fruit transcripts used in the search for SSRs. **Table S2**: 122 transcript sequences from Passiflora edulis that were used for designing the primers. **Table S3**: Putative gene function of passion fruit transcripts containing SNPs (A) and SSRs (B).

## Abbreviations

AFLP: amplified fragment length polymorphism
QTL: quantitative trait loci
RAPD: random amplified polymorphic DNA
RGA: resistance gene analog
SNP: single nucleotide polymorphism
SSH: suppression subtractive hybridization
SSR: simple sequence repeats
TRAP: target region amplification polymorphism
*Xap*: *Xanthomonas axonopodis* pv. *passiflorae*
